# Fracture resistance of CAD-CAM ceramic versus PFM endocrowns in cariously extracted mandibular Teeth: An *in vitro* study

**DOI:** 10.6026/973206300212643

**Published:** 2025-08-31

**Authors:** Arti Shyam, Alka Gupta, Harsh Chansoria, Mukesh Soni, Kritika Kar, Shivani Gupta

**Affiliations:** 1 Department of Prosthodontics and Crown & Bridge, Government College of Dentistry, Indore, Madhya Pradesh, India; 2 Department of Orthodontics and Dentofacial Orthopaedics, Sri Dharmasthala Manjunatheshwara College of Dental Sciences, Dharwad, Karnataka, India

**Keywords:** Adhesive bonding, CAD-CAM, endocrown, fracture resistance, zirconia

## Abstract

The fracture resistance of Computer-Aided Design/Computer-Aided Manufacturing (CAD-CAM)-fabricated porcelain-fused-to-metal (PFM) and all-ceramic (monolithic zirconia) endocrowns in cariously extracted mandibular premolars and molars. Sixty root canal-treated teeth were restored and tested using standardized protocols. Fracture resistance was measured with a Universal Testing Machine. Results showed significantly higher resistance in zirconia endocrowns across all tooth types. Thus, we show the use of zirconia as a more durable restorative material for posterior teeth with structural compromise.

## Background:

One of the primary challenges in reconstructive dentistry lies in the restoration of endodontically treated teeth with severely compromised coronal hard tissue [1]. Rehabilitating such extensively damaged teeth-whether due to advanced dental caries or traumatic injury-requires meticulous planning, innovative restorative strategies and precise clinical execution [2]. Unlike changes in dentin composition, structural compromise caused by caries, trauma, or material degradation undermines the tooth's integrity, thereby reducing both stiffness and fracture resistance [3]. In particular, this diminished structural integrity-when compared with vital, non-endodontically treated teeth-increases the risk of microleakage at restoration margins and crown fractures [4]. Furthermore, the loss of pulpal vitality limits sensory feedback, making it more difficult to detect early signs of structural compromise and increasing susceptibility to fracture in non-vital teeth [5]. Traditionally, severely damaged teeth have been restored using post-and-core systems in combination with crowns. However, these conventional approaches often weaken the remaining tooth structure, heightening the risk of root fracture and jeopardising the long-term prognosis of the restoration [6]. Contemporary restorative approaches emphasize the preservation of as much healthy tooth structure as possible and among these; the endocrown has emerged as a minimally invasive alternative [7]. The concept of the endocrown was first introduced by Pissis in 1995. It is a monolithic restoration that gains retention from both the internal walls of the pulp chamber and the cavity margins, combining macromechanical and micromechanical bonding mechanisms [8, 9]. Endocrowns are particularly advantageous for posterior teeth with short, curved, or fragile roots, as they require less invasive preparation and better conserve the remaining tooth structure than traditional post-and-core restorations.

High-strength ceramic materials, such as zirconia and lithium disilicate, further enhance the fracture resistance of endocrowns, making them suitable for withstanding high occlusal forces in posterior regions [10]. With the advent of Computer-Aided Design/Computer-Aided Manufacturing (CAD/CAM) technology, digital impressions and 3D printing, endocrowns have become a reliable and increasingly utilized solution for restoring endodontically treated teeth with substantial coronal loss [11]. A key benefit lies in the endocrown's design, which spans the pulp chamber and enhances retention compared to conventional crowns that rely solely on the remaining coronal structure. This approach improves both macro- and micromechanical stability, significantly reducing the risk of restoration failure [12]. The success of endocrowns is influenced by several factors, including the extent of residual tooth structure, the design of the preparation, the adhesive protocol and the selection of restorative materials. The mechanical properties of ceramics such as zirconia and lithium disilicate largely determine their performance in endocrown applications [13]. Furthermore, the restoration's design must facilitate proper stress distribution and marginal fit to prevent fractures. The choice of adhesive system also plays a vital role in achieving a durable bond between the endocrown and the underlying tooth structure [14]. Therefore, it is of interest to describe the comparative effect of different restorative materials on the fracture resistance of CAD-CAM ceramic and PFM endocrowns in order to guide clinical decisions for endodontically treated teeth with substantial coronal loss.

## Methodology:

This study aims to evaluate and compare the fracture resistance of PFM and All-Ceramic endocrowns for extracted carious mandibular premolars and molars using the CAD-CAM technique. The specific objectives are to assess the fracture resistance of CAD-CAM PFM endocrowns for extracted carious premolars, molars and premolar-molar (splinted) teeth, assess the fracture resistance of CAD-CAM All-ceramic (Monolithic Zirconia) endocrowns for the same group of extracted teeth and compare the fracture resistance between CAD-CAM PFM and All-ceramic (Monolithic Zirconia) endocrowns across different groups. This study is an *in vitro*, prospective, experimental, comparative and quantitative research project conducted at the Government College of Dentistry, Indore and Madhya Pradesh, India. Fracture resistance testing was carried out at the KAILTECH Test and Research Centre Pvt. Ltd., Indore. Ethical approval was obtained from the institutional ethical committee prior to the commencement of the study. The specimens selected for this study include extracted mandibular premolars, molars and premolar-molar combinations that have undergone root canal treatment. These extracted teeth serve as the base samples for creating the endocrown restorations using both PFM and All-ceramic (Monolithic Zirconia) materials (Figure 1 - see PDF). Materials tested in the study include metal alloy ingots for PFM from Radiant Metals and Alloys Pvt., ceramic powders for PFM from Ivoclar (IPS Classic Dentin) and Monolithic Zirconia from UPCERA. The methodology for preparing these specimens involves various materials and tools, such as face masks, sterile gloves, dental plaster, assorted RCT files, guttapercha points and composite restoration material, all sourced from reputable manufacturers. The equipment and accessories used in this study include a universal testing machine (Deepak Polyplast, model DUTT-101), a digital extraoral lab scanner (MeditIdentica Depot), CAD software (Exocad® Germany), milling machines for both Monolithic Zirconia and PFM (GTR DX5), a ceramic furnace (VITA V60 I-line porcelain furnace), a zirconia sintering machine (BLOOMDEN) and an IOPAR device (GOMEX wall-mounted dental X-ray system). The tooth preparation for the study utilized a range of materials, including dental burs for tooth preparation, specifically Mani diamond burs of different types (*e.g.*, round bur-BC 31, TR 12, TF 12) and Airotor handpieces (NSK, Europe GmbH). Additionally, PFM CAD CAM wax blanks (MAARC CAD CAM milling wax) were used to design and mill the restorations. RMGIC Cement Fusion Ultra D/C was used to bond the endocrowns to the prepared teeth. This technique ensures that the fracture resistance of both PFM and All-Ceramic endocrowns is objectively evaluated, thereby providing valuable information for comparing the two materials in restoring endodontically treated teeth. The design, materials and methods of the research are specifically chosen to provide a comprehensive assessment of fracture resistance, offering informative insights to the discipline of restorative dentistry. Using the CAD-CAM method, this study follows several phases aimed at assessing and comparing the fracture resistance of PFM and All-Ceramic endocrowns for excised carious mandibular premolars and molars. Selection and storage of the specimens are the first steps in the approach. This work focused on sixty extracted mandibular premolars and molars. Every specimen showed carious lesions exactly on the occlusal surface and intact proximal surfaces. Not discussed at all were past repairing techniques, abrasion, or degradation. To preserve the specimens, they were painstakingly cleaned with hydrogen peroxide and then kept in distilled water at room temperature. To guarantee the tooth's long axis ran perpendicular to the plaster block, each specimen was then individually set on it. This mounting kept the specimens steady during subsequent treatments. Root canal treatment was then performed on the extracted tooth specimens. This involved biomechanical preparation, which involved creating an access cavity to reach the pulp chamber and canals. Endodontic files were used and irrigation solutions were employed to disinfect the inner canal space. After cleaning, the canals were dried and filled with sealer and obturated with guttapercha to seal the canals. A radiograph was taken of each obturated specimen to ensure proper filling. Tooth preparation followed, adhering to a standardized protocol using the Shofu endocrown and bridge tooth preparation kit. The specimens were divided into two groups based on the materials used for fabrication. Group I consisted of PFM endocrowns and Group II consisted of all-ceramic (monolithic zirconia) endocrowns. The tooth preparation for Group I was done with a buccal shoulder and lingual chamfer finish line, while Group II was prepared with a deep chamfer finish line on both the buccal and lingual surfaces.

For the fabrication of the endocrowns, extraoral scanning was carried out. The prepared teeth mounted on plaster blocks were scanned using a digital extraoral scanner (MeditIdentica Depot). The digital impression was taken and the Standard Tessellation Language (STL) file of the scanned models was obtained. The endocrowns were designed using EXOCAD software and STL files were created. A cement space thickness of 30 µm was defined for the design. The next step was the fabrication of the endocrowns. For the monolithic zirconia endocrowns, the STL file was imported into the CAM software. A pre-sintered zirconia block (UPCERA) was placed into the milling machine and the zirconia was milled into the shape of the endocrown. After milling, the zirconia was sintered in a high-temperature furnace (1350-1530°C), followed by polishing with an acrylic finishing and polishing kit. For the PFM endocrowns, the STL file was also imported into the CAM software and a CAD/CAM milling wax blank (MAARC CAD CAM milling wax) was placed into the milling machine. The fabricated wax pattern underwent standardized casting procedures to create the metal substructure (Radiant Metals & Alloys Pvt.), which was then layered with dental porcelain and fired in a high-temperature furnace. The prosthesis was finished and polished using a ceramic finishing and polishing kit. Once the endocrowns were completed, cementation was carried out. The inner surfaces of the PFM and all-ceramic endocrowns were evaluated for any voids, bubbles, or marginal inadequacies. Resin-modified cement (Prevest Ultra Fusion D/C Intro Pack Luting Resin Cement) was used for cementation. The inner surface of the endocrown was cleaned and dried before being luted with the cement. The endocrown was placed on the prepared tooth with finger pressure to maintain constant pressure after the initial set and excess cement was removed using an explorer. Finally, the mechanical testing of the specimens was performed. After cementation, all specimens were mounted on acrylic resin blocks for testing. The fracture resistance of the cemented specimens was measured using a Universal Testing Machine (Deepak Polyplast, MODEL NO. DUTT-101). The specimens were placed on a metal base at a 135 ° angle to the long axis of the tooth, with a stainless-steel ball (3 mm in diameter) placed over it. The load was applied in a vertical direction along the long axis of the tooth, ensuring that the load contacted the central fossa before being applied. The specimens were stabilized to prevent rotation and the fracture origin was observed at the point of loading. The force required to fracture the specimens was measured in Newtons (N), with the fracture resistance calculated as the maximum load N.m. This testing provided quantitative data for comparing the fracture resistance of PFM and all-ceramic endocrowns.

The data obtained in this study were subjected to statistical analysis with the assistance of a statistician. The data were compiled systematically and organized into a master table, from which individual tables and graphs were generated for presentation purposes. The statistical procedures were carried out in two main steps: data compilation and statistical analysis. Data were entered into an Excel spreadsheet and analyzed using SPSS (Statistical Package for the Social Sciences) version 25.0. The Kolmogorov-Smirnov test was performed to check for probability distribution and it was found that the data followed a normal distribution. Descriptive statistics were calculated as mean ± standard deviation and inter-group comparisons were made using the independent test and One-way ANOVA. Post hoc analysis was conducted using Tukey's test, with a p-value of <0.05 considered statistically significant. To analyze the data, various statistical measures were used. The mean was calculated by dividing the sum of all data values by the number of observations. At the same time, the standard deviation (SD) was used to describe the variability of the dataset. The standard error of the mean (SEM) was calculated by dividing the SD by the square root of the sample size. The Student t-test was applied to compare two independent means, ensuring that the data were normally distributed and the two samples had the same variance. For comparing more than two groups, One-way ANOVA was performed, followed by post hoc tests using Tukey's Honestly Significant Difference test to determine which group means were significantly different from one another. The p-value was used to indicate the level of significance, with p > 0.05 considered not significant, p <0.05 considered important and p < 0.01 and p < 0.001 indicating highly substantial and very highly significant differences, respectively. The null hypothesis for this study posited that there would be no significant difference in the fracture resistance of PFM and All-Ceramic (Monolithic Zirconia) endocrowns. Conversely, the alternative hypothesis suggested that there would be a substantial difference between the two materials in terms of their fracture resistance when used for prosthodontic restorations.

## Results:

The fracture resistance for the PFM and All-Ceramic (Monolithic Zirconia) endocrowns was measured across three groups: premolar, molar and premolar-molar endocrowns. The fracture resistance values for the PFM endocrowns, where the fracture resistance of premolar endocrowns (Group 1.1) ranged from 1029 to 1496 N, molar endocrowns (Group 1.2) from 1556 to 2502 N and premolar-molar endocrowns (Group 1.3) from 2160 to 2689 N. Similarly, the fracture resistance values for the All-Ceramic endocrowns, where premolar endocrowns (Group 2.1) showed a range from 2698 to 3049 N, molar endocrowns (Group 2.2) ranged from 2387 to 2672 N and premolar-molar endocrowns (Group 2.3) varied from 2589 to 3126 N. Statistical analysis using one-way ANOVA revealed significant differences in fracture resistance between the groups within the Zirconia endocrowns. The premolar endocrowns had a mean of 1241.0 N (Group 1.1), molar endocrowns had a mean of 1940.1 N (Group 1.2) and premolar-molar endocrowns had a mean of 2397.4 N (Group 1.3) with a significant p-value of <0.001. Similar results for the Zirconia endocrowns, with the premolar endocrowns (Group 2.1) having a mean of 2830.6 N, molar endocrowns (Group 2.2) a mean of 2517.0 N and premolar-molar endocrowns (Group 2.3) a mean of 2876.5 N. The differences were again statistically significant (p <0.001). A comparison of fracture resistance between the PFM and Zirconia groups for premolar endocrowns showed that Zirconia (Group 2.1) had a significantly higher mean fracture resistance (2830.6 N) than the PFM group (Group 1.1), with a mean of 1241.0 N (p-value <0.001).

Similarly, for molar endocrowns, Zirconia (Group 2.2) had a mean of 2517.0 N, which was significantly higher than the PFM group (Group 1.2) with a mean of 1940.1 N (p-value <0.001). Table 1 (see PDF) provides an inter-group comparison, highlighting the statistical significance between the different endocrown materials and configurations. The p-values for most comparisons, such as between Group 1.1 and Group 2.1 (p-value <0.001), indicate significant differences, except for the comparison between Group 1.3 (PFM Premolar-Molar Endocrown) and Group 2.2 (Zirconia Molar Endocrown), which was not significant (p-value = 0.761). In terms of intra-group comparisons (Table 1 - see PDF), significant differences were found between the PFM and Zirconia groups, with p-values for the majority of comparisons being less than 0.05, indicating that the fracture resistance of the Zirconia endocrowns was consistently greater than that of the PFM endocrowns. Finally, the graphical representation of the fracture resistance across the different groups, shown in Figure 2 (see PDF), visually supports the statistical findings, demonstrating higher fracture resistance in the Zirconia groups compared to the PFM groups. The p-values indicate the statistical significance of the differences between the groups. Significant differences are marked with an asterisk (*).

## Discussion:

Endodontic treatments, which are routinely performed in dental practices, often necessitate the removal of substantial tooth structure, potentially compromising the tooth's overall strength and long-term durability [15]. This structural weakening is particularly evident in posterior teeth, which are more susceptible to fractures following root canal therapy. Due to their anatomical and functional demands, these teeth typically require protective cusp coverage. In such scenarios, endocrowns have emerged as a reliable restorative option, especially when there is significant coronal loss, limited interocclusal space, or a reduced clinical crown height. Moreover, they are particularly advantageous in cases where conventional adhesive restorations may not be feasible [8]. Unlike traditional crowns, which require extensive tooth reduction and post-placement within the root canal to enhance retention, endocrowns employ a conservative and innovative approach. Characterized by a butt-joint margin at the occlusal surface, the restoration extends into the pulp chamber, securing macromechanical retention without intruding further into the root canal system [14]. This design eliminates the need for posts, thereby preserving more natural tooth structure and reducing the risk of root fractures commonly associated with post placement. The minimally invasive preparation and simplified clinical protocol of endocrowns also reduce both chairside and operative time, making them an ideal solution for restoring severely damaged posterior teeth [16]. Introduced by Pissis in 1995, the endocrown challenged traditional post-and-core concepts by reconstructing non-vital teeth using a monolithic, one-piece restoration composed entirely of ceramic or composite material. This design engages the pulp chamber and, to a limited extent, the root canal, employing a small endo-core for added retention while avoiding traditional post systems. The result is enhanced structural integration and stability, offering a conservative yet robust alternative for restoring endodontically treated teeth [17]. Endocrowns achieve retention through contact with the internal walls of the pulp chamber and cavity margins, complemented by micromechanical bonding via adhesive cementation. Initially proposed as a replacement for metal post-and-core systems, the monobloc approach of endocrowns provides a conservative, efficient and aesthetically pleasing solution for structurally compromised teeth. Their long-term clinical success is closely tied to their ability to withstand functional occlusal forces. Manufacture primarily from materials such as zirconia and porcelain-fused-to-metal (PFM), endocrowns exhibit mechanical properties that contribute significantly to their strength and resistance to functional stressors [18]. PFM crowns have been widely used in restorative dentistry due to their favourable combination of strength and esthetics. The metal substructure provides mechanical stability, while the porcelain veneer mimics the natural appearance of teeth. PFM endocrowns are reported to offer acceptable fracture resistance, particularly in posterior teeth subjected to high occlusal loads. The bonding between the metal and porcelain-achieved through compressive forces, mechanical interlocking and van der Waals interactions-enhances the restoration's durability and performance [19].

In contrast, zirconia has gained increasing popularity owing to its superior mechanical properties, particularly its high fracture toughness. Monolithic zirconia endocrowns, fabricated from a single zirconia block, are well-suited for posterior restorations under significant occlusal stress, as they effectively resist both fracture initiation and propagation [20]. Furthermore, advancements in zirconia formulations have produced high-translucency variants that improve esthetic integration with natural dentition [21]. The fracture resistance of zirconia endocrowns is influenced by several critical factors, including the precision of the restoration's design, the specific zirconia composition and the quality of adhesive bonding and the magnitude of masticatory forces. A thorough understanding of these variables is essential to ensure the long-term success of zirconia-based restorations [22]. The integration of CAD/CAM technology has revolutionized the design and fabrication of endocrowns. Precise digital planning and milling enable superior marginal fit, preservation of remaining tooth structure and improved mechanical performance and durability. Additionally, the use of digital workflows significantly reduces chairside time, thereby improving treatment efficiency and patient comfort [23]. In discussing the role of endocrowns in restoring endodontically treated teeth, it is essential to highlight the findings of Jalalian *et al.* (2024) [24], which explore the significant advantages of endocrowns, particularly in terms of their ability to restore severely damaged posterior teeth. The study emphasizes the structural challenges faced by teeth following endodontic treatment, which often leads to substantial loss of coronal tooth structure, compromising the tooth's strength and increasing its susceptibility to fractures. This is particularly evident in posterior teeth, where the functional demands and occlusal forces are higher. The present work highlights the improved performance of zirconia in this respect by providing significant additional insights into the fracture resistance of PFM and zirconia endocrowns. These findings contribute to the growing body of research on endocrown materials, particularly in terms of their mechanical properties and medicinal efficacy. Zirconia is a consistently preferred choice for dental materials, especially in challenging clinical situations that require minimal tooth preparation due to its remarkable fracture resistance. Future research should examine the long-term performance of these materials in conjunction with factors such as material thickness, cementation techniques and the impact of occlusal stresses on the lifetime of restorations.

## Conclusion:

Zirconia endocrowns exhibit better fracture resistance than PFM endocrowns. Thus, they are a more consistent alternative for treating structurally problematic teeth. These restorations can achieve increased accuracy and strength through the use of CAD/CAM technologies. Through guided material decisions and application tactics, constant research will yield improved long-term therapeutic results.
